# Experimental Estimation of Kinematic Viscosity of Low-Density Air Using Optically Derived Macroscopic Transient Flow Parameters

**DOI:** 10.3390/s25113375

**Published:** 2025-05-27

**Authors:** Tomasz Aleksander Miś

**Affiliations:** Space Research Centre of Polish Academy of Sciences, 00-716 Warsaw, Poland; tmis@cbk.waw.pl or tomasz.a.mis@mailplus.pl

**Keywords:** Roshko, oscillations, camera, optical, parachute, viscosity, stratosphere, near-space

## Abstract

This article presents a novel experimental method of calculation of kinematic viscosity parameter for rarefied/low-density air using the analysis of optically recorded oscillations of the stratospheric balloon mission parachute’s canopy. The parachute behavior was captured by a high-definition optical device in the stratosphere during the re-entry phase, giving the input data for the Roshko and Reynolds numbers, which were used in an adapted formula to determine the kinematic viscosity. The calculated parameter was compared with laboratory literature data, showing good alignment, with any sources of discrepancies indicated and discussed. The canopy-breathing method of determination of kinematic viscosity in rarefied air can be employed for the easy investigation of real atmospheric parameters, helpful in the analysis of atmospheric and ionospheric mass flows and the design and performance verification of various novel types of parachutes and re-entry devices.

## 1. Introduction

Low-air-density air properties are crucial for the design and performance verification of high-altitude flight systems, mainly re-entry devices for stratospheric missions and orbital landers [1]. Such systems usually include parachute systems of different compositions [2,3] or autorotation devices; their simulation does not always yield accurate or stable results, which may exhibit statistical nature [4] or large changes in the environmental parameters, despite validly approaching the high-altitude atmosphere as a continuous medium [1]. Experimentally verified air/airflow parameters of the actual atmosphere are therefore crucial to increase the accuracy and stability of such computational solutions, producing results better corresponding to real conditions. The application of such solutions is especially important for high-altitude balloon observation platforms, where the designed physical stability of the setup in rarefied airflow is an important factor allowing an easy comparison with terrestrial measuring equipment, e.g., the LOFAR telescope system, throughout the entire flight.

The design and analysis of such re-entry devices essentially employs rarefied gas dynamics, which is, however, not exclusively limited to aircraft or spacecraft. High-altitude atmospheric mass flows are one of the crucial factors contributing to the formation and evolution of, e.g., subsequent ionospheric layers, which are subjected to different phenomena within the still continuous albeit rarefied air. Such phenomena frequently include, e.g., the formation of the sporadic E ionospheric layer by ion descent from the F layer and the wind shear mechanism [5,6] or layer deformations (tilting, modulation) by atmospheric tides [7] (although exceptions appear [8]), and gravity waves [9,10,11], which were, e.g., reported to rotate the plasma density gradient inside the ionized structures [12] and causing the TIDs—Travelling Ionospheric Disturbances [13]. Other reported more-detailed phenomena include, e.g., the formation of convective rolls-like structures inside the layers [14], large and small-scale layer irregularities and depletion regions (to which also neutral atmospheric motions actively contribute) [15,16] or layers to the decay of which the diffusion mechanisms actively contribute [17]. The chemical aspects of the ionospheric layer formations, including the ion transfer from highly positioned layers to the lower ones, also play an important part in their formation and evolution [5,16,17,18]. Accurate estimation and simulation of such phenomena, regardless of their scale (large, local, periodic, ephemeral, macroscopic, ionic), require an accurate definition of the actual atmospheric parameters at these altitudes (typically > 90 km) in order to produce results of higher quality, comprising not only the ion- or plasma physics-related factors, but also fluid mechanics factors.

Air pressure and temperature are often easily measured in flight campaigns using stratospheric balloons or sounding rockets as flight vehicles/research platforms. Parameters requiring the measurement using mostly indirect methods—e.g., the dynamic or kinematic viscosity—are physically more difficult to carry out, especially on board a vehicle which transverses the majority of the dense part of the atmosphere and experiences varying dynamic flight conditions. Analytically, it is possible to attempt the derivation of such parameters from, e.g., the kinetic gas theory, which often incorporates other variables, such as the particle’s dimensions and molar volumes, temperature or sublimation energy [19]—such derivation would, however, eventually require an experimental confirmation. In this article, one of such experimental methods is analyzed—a method of calculation of the stratospheric air’s kinematic viscosity using an indirect method of the optical measurements of the parachute canopy’s breathing due to transient flow in the beginning of the balloon mission’s re-entry phase (after the balloon burst at the maximum altitude), later used as an input to the calculations using the Roshko number. The results are then compared with the literature (laboratory) model of the air viscosity altitude profile and discussed, showing the advantages of the presented method and the possibilities of its employment.

## 2. Parachute Systems’ Dynamics

For space applications, the majority of the design aspects of the parachutes revolve around the requirement of reducing the velocity of the spacecraft in low-density atmosphere at transonic or supersonic conditions, including foreign planet atmospheres (e.g., Mars) [20]. The naturally occurring oscillations of the parachute systems at these velocities can be distinguished into material-related (low frequency, mainly at the edge of the canopy) and wake-related (high frequency) [3], with the system’s tethers also contributing to the formation of these oscillations [21]; generally, the mechanical properties of the system—such as the spring constants of the fixing points—are strongly affected by the oscillations [22]. The vortex shedding from the parachute during flight causes its canopy to move inwards and outwards (the previously mentioned ‘canopy breathing’) [23], causing the lift and drag forces’ pulsations [20], with the drag coefficient and the vortex shedding frequency decreasing with the increasing Mach number [24]. The asymmetrical shedding of the vortexes also often causes the swinging of the entire parachute, yet the variation of the position of the vortex center is found the same for super- and subsonic flows [25]. The oscillations of the canopy can be effectively reduced by the placements of secondary objects in front of the parachute, e.g., a ring structure [26]; similar effects for lower Reynolds numbers could be obtained by the placement of hairy flaps on the blunt body in the region of the wake’s creation [27].

The main difference between these parachute analyses and the case described in this article is the subsonic flow around the parachute. While in space missions, the parachute is gradually opened (‘blooming’) at the precisely defined altitude, for a near-space stratospheric balloon mission, the device, positioned between the gondola and the balloon itself, stays deployed during the entire flight and starts to open immediately after the re-entry phase begins (e.g., after the cutoff from the balloon or after the latex balloon bursts) [28]. Despite moving in the subsonic velocity region, the parachute, however, experiences similar phenomena to those described above for supersonic velocities—for the initial phase of the re-entry, the parachute is unable to operate properly, rhythmically opening and closing its canopy; this behavior ends at the altitudes below 15–12 km, where the canopy remains constantly open and creates and effective drag force, slowing down the entire flight train.

### 2.1. Experimental Observations

**Figure 1:**
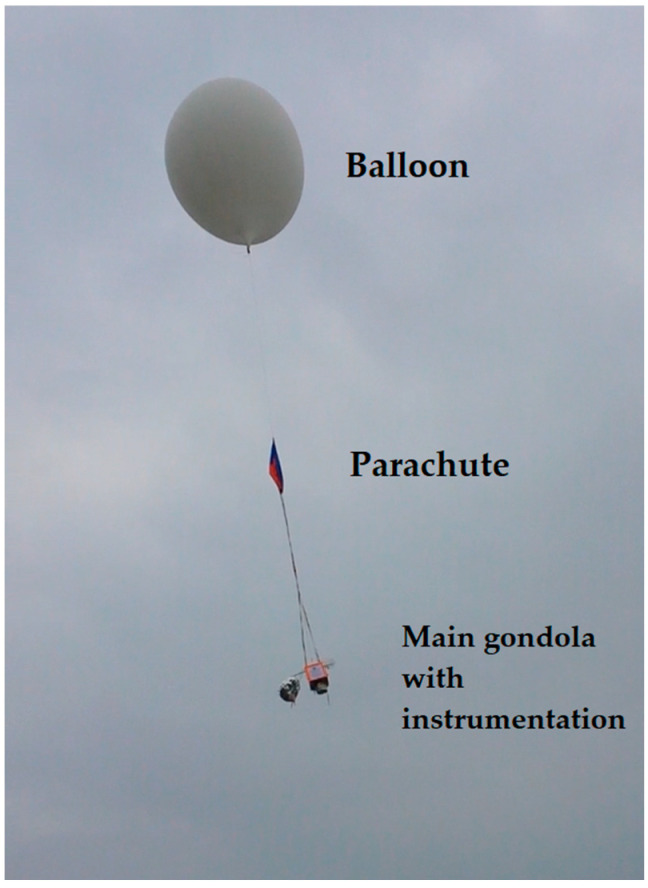
The flight train of the stratospheric balloon mission after launch. Atop the main gondola, the ultra-wide-angle lens camera monitored the movement of the parachute (Figure 2).

**Figure 2:**
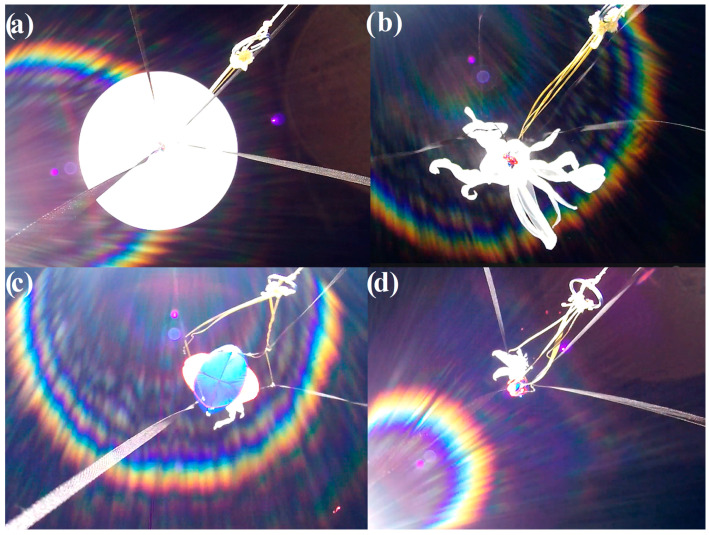
The subsequent phases of the beginning of the re-entry of a stratospheric balloon mission; (**a**) latex balloon at the maximum altitude, (**b**) balloon burst, (**c**) parachute canopy fully opened, (**d**) parachute canopy fully closed.

**Figure 3:**
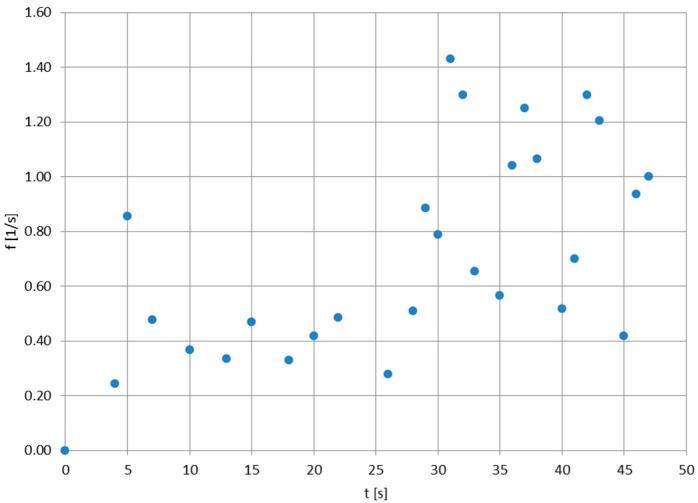
The values of the canopy oscillation frequency for the beginning of the re-entry phase of the stratospheric balloon mission; value accuracy: ±0.0196 Hz. After 30 s of re-entry, the canopy breathing increased and oscillated above 1 per second, with the deviations most probably caused by the canopy’s moving tethers.

The oscillating behavior of the stratospheric parachute can be recorded using an optical sensor placed upwards straight below it, which is a rarely encountered technical setup—therefore, a limited amount of raw data are available for analysis. In this article, the canopy breathing used for analysis was recorded on 18 April 2015 by a HackHD digital camera with ultra-wide-angle lens (120°) with a hemispherical projection, placed on the top of a balloon gondola, launched by the Students’ Space Association of Warsaw University of Technology’s Balloon Division from Goczałkowice-Zdrój in southern Poland, reaching the maximum altitude of 25,300 m [29]. The parachute canopy was of the hemisflo type [20], having the approximate maximum cross-section diameter D of 0.85 m (reduced from nominal dimensions by the parachute tethers, affixed to a 0.3 m × 0.3 m × 0.3 m cubic gondola). Figure 1 shows the mission’s flight train structure after the launch; Figure 2 presents the mosaic of still frames from the camera, showing the latex balloon before burst and the positions of the parachute during the beginning of the re-entry phase. It can be seen that the canopy completely opens and completely closes; the frame-by-frame tracing of the movement of the canopy (e.g., the maximum opening moments) in time yielded the canopy pulsation frequencies, presented in Figure 3. The time scale for these values covers the acceleration of the flight train after the balloon burst to a maximum vertical velocity, which is then slowly decreasing, as the drag force generated by the parachute increases. The accuracy of the readings of the full canopy breathes from the film frames was 0.01 s, yielding the breathing frequency accuracy of ±0.0196 Hz.

**Figure 4:**
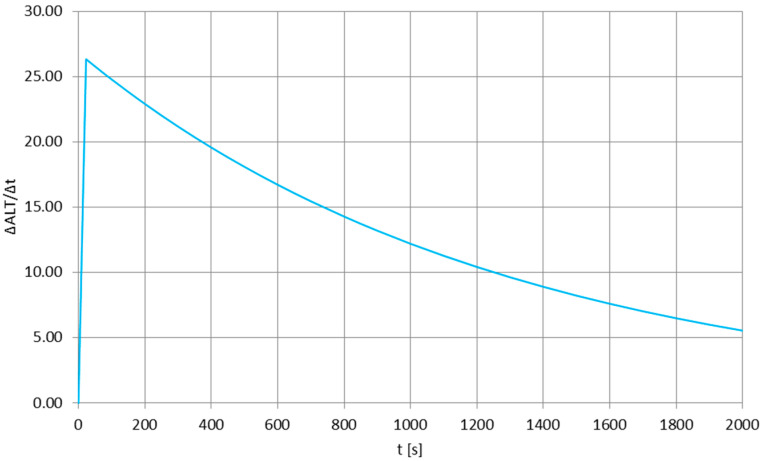
The vertical velocity profile of the stratospheric balloon mission after the balloon burst at maximum altitude.

**Figure 5:**
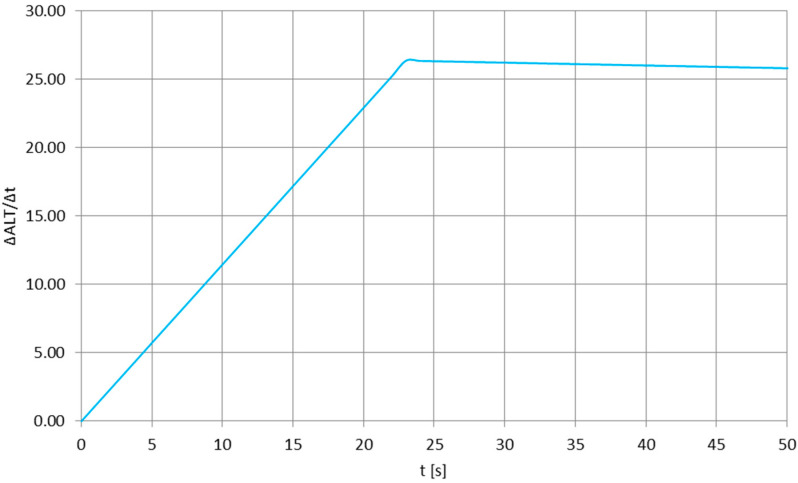
The vertical velocity of the stratospheric flight train during the first 50 s after the balloon burst, to be compared with Figure 1.

Figure 4 presents the experiment-based [29,30,31] vertical velocity profile of the stratospheric balloon mission having a long antenna flight train, with Figure 5 enlarging the function for the first 50 s of the re-entry phase, to be compared easily with time scale in Figure 2. It can be noticed that the first part of both plots shows the rapid acceleration of the flight train due to very low air density and lack of a substantial aerodynamic drag—this short, initial re-entry phase is absent for, e.g., tropospheric descents [32]. Direct comparison of Figure 2 and Figure 5 shows that the quasi-constant oscillation frequency of the parachute’s canopy is observed for the re-entry phase after the rapid acceleration, when the descend velocity is nearly constant. In the time scale of the oscillations (one oscillation every one or more seconds), there are no significant variations in the descend velocity—therefore, the pulsations of the drag force generated by the parachute are nearly nonexistent and the canopy oscillations are essentially related to the forces generated by the vortexes descending from its sides (e.g., as in [33], but for lower Reynolds numbers), moving the canopy until the air density becomes significant enough to create a drag force keeping the canopy stable.

### 2.2. Theoretical Model of Viscous Stratospheric Flow

**Figure 6:**
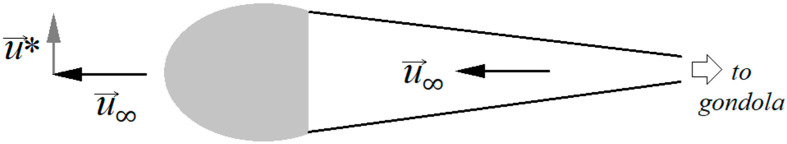
Basic flow schematic for the parachute in the stratospheric re-entry phase.

Figure 6 presents the basic flow schematic of the parachute during the re-entry phase of the stratospheric flight. Behind the canopy, the undisturbed flow velocity vector ${\vec{u}}_{\infty }$ is accompanied by the perpendicular and averaged [34] vector of velocity of the disturbed flow $\vec{u^{*}}$, which can be assumed as responsible for the flow instability and the breathing of the canopy. The flow behind the canopy for the stratospheric atmospheric conditions remains, however, not entirely turbulent—the flow is dominated by viscosity [35,36,37] and the disturbed, non-laminar layer forms an envelope around the object, moving along with it [38]; the drag force remains proportional to the cross-section diameter, not the cross-section area [38].

The value of the disturbing velocity vector $\vec{u^{*}}$ can be shown as proportional to [39]:

(1) $$\left|\vec{u^{*}}\right|\sim \frac{F}{\rho u^{2}D^{2}}$$

where *F* [N] is an aerodynamic force (drag, lift), *ρ* [kg/m^3^] is the air density, *u* [m/s] is the descent velocity (equal to |${\vec{u}}_{\infty }$|) and *D* [m] is the approximated diameter of the canopy. Since the drag force was not experiencing recognizable pulsations, the force *F* shall in this case can be attributed to the lift force causing the breathing of the canopy. Since the air density *ρ* remains diminutive, the main factor/variable driving the mechanism behind the *F* force is the descent velocity *u*—which can be noticed in Figure 3, where the maximum (in this experiment) number of oscillations is observed for the maximum velocity during the re-entry phase (Figure 5).

Since the flow for the aforementioned atmospheric conditions remains essentially viscosity-dependent, a solution can be formed to combine the observed oscillations of the parachute’s canopy and the viscosity of the surrounding air, treating other factors as constants or variables of known behavior in time or in altitude. The main part of the solution is the Roshko number *Ro*, dependent on the air’s kinematic viscosity *ν* [m^2^/s], the diameter *D* of the object and the frequency *f* [Hz] of the shedding of the vortexes [40,41], here attributed to the frequency of full breathing of the canopy:

(2) $$Ro=f\frac{D^{2}}{\nu }$$

The investigations employing the Roshko number in its basic form shown above were, however, usually limited to cylinders or spheres [42,43], deriving continuous functional relations between the Roshko and Reynolds numbers. For the purpose of the canopy breathing experiment analysis (the canopy as an object essentially experiencing alternating shedding of vortexes in the airflow around it, similarly to cylinders and spheres), one of such combining formulas [43], derived for higher Reynolds numbers *Re* (from 260 to 360), can be employed and modified:

(3) $$a\cdot Ro=-48.2+0.391\cdot Re-0.0036\cdot Re^{2}$$

(4) $$Re=\frac{uD}{\nu }$$

where the newly introduced coefficient *a* [-] is a proposed simplified way to refer or recalculate the Roshko number for a sphere to the Roshko number for a parachute’s canopy, approximated as a half-sphere, only if the experimental flow conditions remain comparable and no other variables that those contained in the basic Roshko number Formula (1) are considered (the numbers are unit-less). The coefficient can be defined as:

(5) $$a=\frac{C_{x\;P}}{C_{x\;S}}\;\wedge \;Re_{C_{x\;P}}=Re_{C_{x\;S}}$$

where the *C_x i_* [-] denote the *i*-th drag coefficients, with subscript *P* for parachute and *S* for the half-sphere. Usually, the drag coefficients for various objects of different shapes are available for high Reynolds numbers, corresponding to conventional aircraft dynamics, with a possible limited mean of re-calculation for other *Re* values using the von Kármán approach to the boundary layer kinematics [44]. For the *a* coefficient, however, this Reynolds number re-calculation would eventually be shortened, rendering the a coefficient constant—but with the assumption that the Reynolds numbers for both used drag coefficients were equal. For the case in this analysis, the value of *a* would be equal to 7.2 [44].

From Formula (3) with parameters (2) and (4) included and expanded, the real solution for the kinematic viscosity *ν* can be found—calculated using Mathematica:

(6) $$\nu =\frac{\sqrt{{\left(5000aD^{2}f-1955uD\right)}^{2}-17352000uD}-5000aD^{2}f+1955uD}{482000}$$

The square root in the numerator can be used to determine the ranges of applicability of the formula; for the flow velocity *u*:

(7) $$u\geq \frac{8}{391}\left(125aDf+\frac{3}{D}\sqrt{1205}\right)\;\wedge D>0$$

or for the canopy breathing frequency *f*:

(8) $$f\geq \frac{24\sqrt{1205}\frac{1}{D}+391u}{1000aD}\;\wedge \left\{\begin{array}{c} u\in R_{+}\\ a>0\\ D>0 \end{array}\right.$$

**Figure 7:**
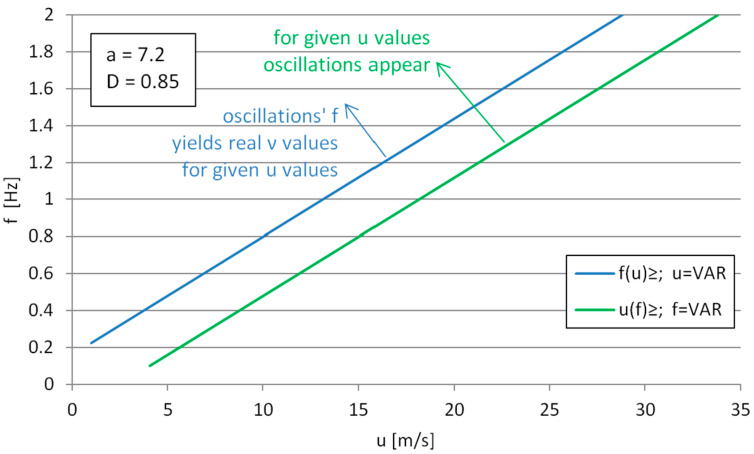
The velocity- and canopy-breathing frequency ranges of applicability for the solution on the kinematic viscosity.

The total range of applicability of the formula can be described as the common part (logic conjunction), or product of sets consisting of upper half-planes limited by the Functions (7) and (8). Figure 7 shows the range plotted in the *u*-*ν* coordinate system, with inequality (7) plotted as a green line and inequality (8) plotted as a blue line. It can be seen that above the *f*(*u*) curve, the observed oscillation frequencies (for the corresponding flow velocities) are able to render the square root in the numerator of (6) real, giving the real (physically possible) solution to the kinematic viscosity.

## 3. Literature Viscosity Models and Comparison with Experimental Method

The results obtained from Formula (6) can be verified against the existing data on viscosity (mainly dynamic viscosity, which yields kinematic viscosity after division by the air density).

The dynamic viscosity for the air can be found as mainly temperature-dependent and weakly pressure-dependent [38,45]. Knowing the interpolated relationship between the dynamic viscosity µ [Pa·s] and the air pressure p [Pa] using data points from [45]:

(9) $$\mu \left(p\right)=17.41-77.58\cdot p^{-0.86}$$

and incorporating it into the interpolated formula of dynamic viscosity in temperature T [K] (data points from [45]), the two-variable solution becomes:

(10) $$\mu \left(T,\;p\right)=0.1368\cdot T^{0.8627}\cdot \left(1.006-4.514\cdot p^{-0.8614}\right)$$

**Figure 8:**
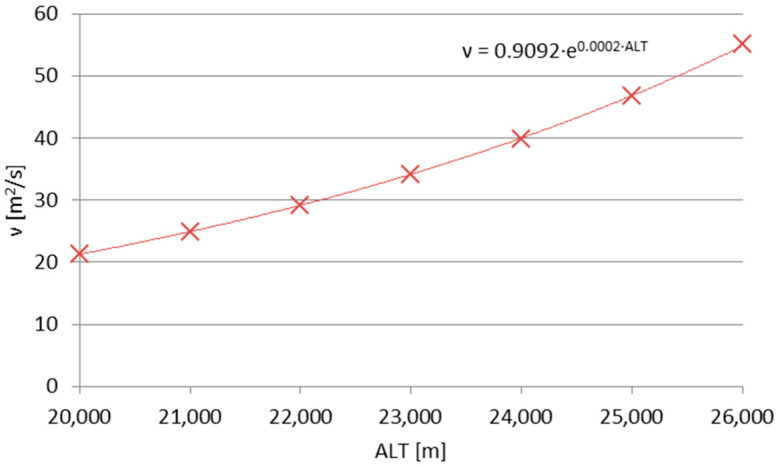
The kinematic viscosity function of altitude with its new interpolation, based on data from Bretsznajder [45], Prandtl [38] and Pankratov [1].

The two-variable formula in Formula (10) can employ the pressure profile for high-altitude atmosphere, including the division by the discrete air density values for subsequent altitude values [1] to obtain the kinematic viscosity. The resulting kinematic viscosity profile for the altitudes considered in this article’s balloon mission is plotted in Figure 8.

**Figure 9:**
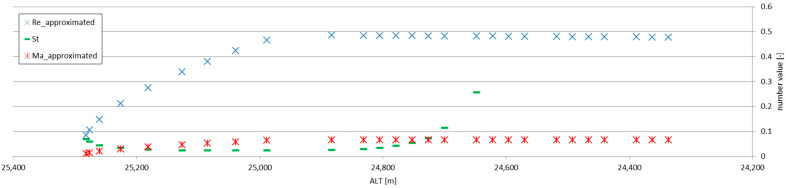
Reynolds, Strouhal and Mach numbers calculated for the stratospheric re-entry experiment with breathing parachute canopy (with the Re and Ma numbers employing approximated descent velocity).

The direct comparison between the results of Formulas (10) (literature; laboratory experiments) and (6) (stratospheric flow) immediately yielded the difference in magnitudes of the values, which can be attributed to the difference of magnitudes between the Reynolds numbers for which the basis (3) of Formula (6) was created and for the actual stratospheric flow. Figure 9 shows the Reynolds (3), Strouhal $St=f\frac{D}{u}$ and Mach $Ma=u{\left(\kappa \frac{p}{\rho }\right)}^{-0.5}$ numbers, plotted together for the maximum altitude ranges considered in this analysis (for the Mach number, *κ* ≈ 1.4 ≈ const.; for the Reynolds number, *ν* = 46 m^2^/s). The flow is essentially subsonic and of very low Re numbers. If the values calculated using the Formula (6) are multiplied by 10^3^—which can be attributed to the compensation of the difference between the orders of magnitude of Re number values between the experiments forming (3) and the actual stratospheric flow (as in Figure 8)—the values calculated using the Formula (6) become comparable with those from laboratory experiments.

**Figure 10:**
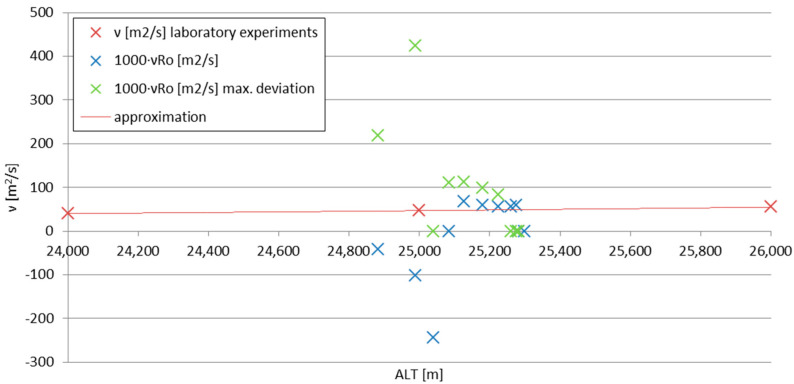
The comparison of viscosities calculated from laboratory experiments [1,38,45] (ν) and the stratospheric breathing-canopy experiment (νRo); ‘max. deviation’ indicates the difference of the readout of the canopy breathing from the film frames at the level of 0.01 s.

The comparison is plotted in Figure 10. For the experimental formula, the maximum deviation data set is included—for the canopy breathing frequencies different by 0.0196 Hz, as mentioned in Section 2.1. It can be noticed that for the transient velocity (below 25.1 km), until the maximum descent velocity is reached, the kinematic viscosity calculation gives results significantly different from the laboratory experiments; when the velocity becomes stabilized, so does the calculated kinematic viscosity. The course of the accurate set of values from the experimental formula aligns well with the theoretical curve, having a small (~10^0^ %) offset; the data set with maximum canopy breathing frequency deviation included stabilizes itself on lower altitudes and reaches the viscosity values a few times larger—this indicates that the accuracy of the readout of the canopy breathing from subsequent film frames must fall below 0.01 s for acceptable accuracy.

The currently employed scaling coefficient can be further attributed to the differences between the experimental (as in the initial Formula (3)) and actual stratospheric Reynolds numbers, altering kinematic viscosity Formula (6) to a more precise form:

(11) $$\nu =\frac{Re_{exp}}{Re_{strato}}\frac{\sqrt{{\left(5000aD^{2}f-1955uD\right)}^{2}-17352000uD}-5000aD^{2}f+1955uD}{482000}$$

where the indexes *exp* and *strato* refer to experimental/laboratory source for (6) and the stratospheric conditions, respectively.

## 4. Conclusions

This article presents an experimental attempt to derive the air’s kinematic viscosity for rarefied atmosphere using the aerodynamic relation based on the Roshko and Reynolds numbers. The parachute’s canopy breathing frequency has been taken directly from the camera-recorded canopy breathing from a stratospheric balloon mission. The results from the developed formula were compared to kinematic viscosity profiles composed from existing data from laboratory experiments and generalized pressure/temperature profile for rarefied atmosphere. The developed formula, after the correction aiming to incorporate the difference between the Reynolds numbers, is shown as similar to the laboratory experiments if the accuracy of the readout of the subsequent canopy breathes is maintained below 0.01 s. Further experimental flight and laboratory-based validation of the formula is possible, with further detailing of the Reynolds number scaling coefficient introduced in Formula (11); the formula, however, describes a novel idea for the determination of the air’s kinematic viscosity in situ, using mandatory flight train components. Such experimental measurements—further stratospheric missions with refined parachute-oriented image recording systems (increasing the accuracy) and parachute canopies of different sizes (exploring the formation and stability of the vortexes)—shall be useful for the determination of actual low-density air kinematic viscosity, not only for the parachute design and performance planning, but also for the analysis or air/ion transfer within the rarefied atmosphere. This remains crucial for the accurate description of the formation and evolution of, e.g., the ionospheric layers from the perspective of fluid mechanics. Another mean of expanding the presented research could be linked to re-entry vehicles aimed for different gaseous celestial bodies of the solar system—the parachute operation recordings from the, e.g., Mars, Venus, Jupiter, Saturn or Titan entries, used as an input for the analysis presented in this article, could deliver additional information on the atmospheric composition and atmospheric dynamics on these worlds.

## Data Availability

Experimental data (camera recordings, etc.) can be made available upon request.
